# Genetic architecture of maize kernel row number and whole genome prediction

**DOI:** 10.1007/s00122-015-2581-2

**Published:** 2015-07-19

**Authors:** Lei Liu, Yanfang Du, Dongao Huo, Man Wang, Xiaomeng Shen, Bing Yue, Fazhan Qiu, Yonglian Zheng, Jianbing Yan, Zuxin Zhang

**Affiliations:** National Key Laboratory of Crop Genetic Improvement, Huazhong Agricultural University, Wuhan, 430070 China

## Abstract

*****Key message***:**

**Maize kernel row number might be dominated by a set of large additive or partially dominant loci and several small dominant loci and can be accurately predicted by fewer than 300 top KRN-associated SNPs.**

**Abstract:**

Kernel row number (KRN) is an important yield component in maize and directly affects grain yield. In this study, we combined linkage and association mapping to uncover the genetic architecture of maize KRN and to evaluate the phenotypic predictability using these detected loci. A genome-wide association study revealed 31 associated single nucleotide polymorphisms (SNPs) representing 17 genomic loci with an effect in at least one of five individual environments and the best linear unbiased prediction (BLUP) over all environments. Linkage mapping in three F_2:3_ populations identified 33 KRN quantitative trait loci (QTLs) representing 21 QTLs common to several population/environments. The majority of these common QTLs that displayed a large effect were additive or partially dominant. We found 70 % KRN-associated genomic loci were mapped in KRN QTLs identified in this study, KRN-associated SNP hotspots detected in NAM population and/or previous identified KRN QTL hotspots. Furthermore, the KRN of inbred lines and hybrids could be predicted by the additive effect of the SNPs, which was estimated using inbred lines as a training set. The prediction accuracy using the top KRN-associated tag SNPs was obviously higher than that of the randomly selected SNPs, and approximately 300 top KRN-associated tag SNPs were sufficient for predicting the KRN of the inbred lines and hybrids. The results suggest that the KRN-associated loci and QTLs that were detected in this study show great potential for improving the KRN with genomic selection in maize breeding.

**Electronic supplementary material:**

The online version of this article (doi:10.1007/s00122-015-2581-2) contains supplementary material, which is available to authorized users.

## Introduction

Maize kernel row number (KRN) per ear is one of the most important yield components and is a breeding goal for the improvement of maize inbred lines. A better knowledge of the genetic architecture of KRN is required to establish breeding programs. However, current knowledge about the genetic control of the maize KRN was mainly obtained from genetic assays of inflorescence mutants. For example, *thicktassel dwarf 1* (*td1*) (Bommert et al. 2005) and *fasciated ear 2* (*fea2*) (Taguchi-Shiobara et al. 2001; Bommert et al. 2013) are involved in meristem maintenance. The *ramosa* mutants can increase the indeterminacy of lateral organs, which transforms the determinate spikelet-pair meristems into branches (Bortiri et al. 2006; Gallavotti et al. 2010; Satoh-Nagasawa et al. 2006; Sigmon and Vollbrecht 2010). *Suppressor of sessile spikelets 1* (*Sos1*) controls meristem determinacy to produce single instead of paired spikelets in the inflorescence, thereby decreasing the KRN in the ear (Wu et al. 2009). The dominant *Corngrass1 (Cg1)* mutant encodes two tandem *zma*-*miR156* genes and leads to a small ear lacking an ordered kernel row and unbranched tassel (Chuck et al. 2007).

However, KRN in natural population displays a quantitative variation that is controlled by numerous QTLs. In the last 20 years, more than one hundred QTLs have been identified by linkage mapping (Veldboom and Lee 1994; Austin and Lee 1996; Ribaut et al. 1997; Yan et al. 2006; Lu et al. 2011a; Li et al. 2014). Despite the increasing accumulation of detected QTLs, the genetic architecture of the KRN has yet to be determined. In addition, the usefulness of these QTLs, which have mainly revealed the allelic variation between pairs of parents, is limited in maize breeding (Xu and Crouch 2008). Recently, association mapping is being increasingly used in plants to uncover genetic effects of diverse alleles within a diverse population (Rafalski 2010). However, the population structure and the detection of rare variants are two major challenges for association mapping (Visscher 2008). A powerful approach that combines linkage analysis and association mapping has been developed to uncover the genetic architecture of complex quantitative traits in maize. For example, Brown et al. (2011) identified 36 QTLs and 261 significant SNPs for the KRN in a nested association mapping (NAM) population by a jointing linkage and genome-wide association study (GWAS).

Genome-wide genotyping also permits the improvement of the trait by genomic selection or whole-genome prediction (WGP), similar to that achieved in cattle breeding. This approach shows a great potential for crop improvement (Lorenzana and Bernardo 2009; Jannink et al. 2010). Previous studies have demonstrated that the KRN can be precisely predicted by genome-wide SNPs using an additive model in bi-parent populations (Riedelsheimer et al. 2013; Guo et al. 2013). However, an additive model using hundreds of trait-associated SNPs cannot predict the KRN well in the NAM population (Brown et al. 2011). In this study, we employed an association panel and three designed F_2:3_ populations to detect the loci involved in KRN variation, and used various marker sets (MSs) and training sets (TSs) to predict the KRN of maize inbred lines and hybrids. Our objectives were to dissect the genetic architecture of the KRN by GWAS and linkage mapping and to evaluate the predictability of WGP for the KRN of maize.

## Materials and methods

### Genome-wide association study (GWAS)

Panel 1, which is composed of 513 inbred lines (Table S1, Yang et al. 2011, the detailed information of these lines can be accessed in http://www.maizego.org/Resources.html), was evaluated for the KRN in five environments, Ya’an (30°N, 103°E), Sanya (18°N, 109°E) and Kunming (25°N, 102°E) in 2009 and Wuhan (30°N, 114°E) and Kunming (25°N, 102°E) in 2010, under a randomized block design with two replicates. At harvesting stage, 8–10 ears in each replicate were phenotyped for KRN. The average value of the two replicates was considered as phenotype of a given genotype, and average KRN in each environment was used to calculate the best linear unbiased prediction (BLUP) of KRN by the linear mixed models of the SAS software (SAS Institute Inc.). The average KRN in each environment and the BLUP results were then used for further research. The repeatability was calculated by *H*^2^ = *δ*_g_^2^/(*δ*_g_^2^ + *δ*_e_^2^/nr) (*δ*_*g*_^2^: genetic variance; *δ*_*e*_^2^: error; *n*: number of environments, and *r*: number of replicates).

The MaizeSNP50 Genotyping BeadChip was used for genotyping Panel 1 (Li et al. 2012) and total of 48,962 SNPs with minor allelic frequencies (MAF) ≥0.05 were obtained. Both generalized (GLM) and mixed linear model (MLM) under a controlling population structure (Q) and principal components analysis (PCA) (GLM, GLM + Q, GLM + PCA, MLM, MLM + Q and MLM + PCA) were applied to establish an association between the SNP and KRN using Tassel v. 3.0 (Yu et al. 2006; Bradbury et al. 2007; Zhang et al. 2010). The Meff method was performed using SNPSpD package to estimate the number of independent tests (*M*_eff_) for the 48962 SNPs of Panel 1 (Nyholt 2004; Li and Ji 2005). The stringent significance threshold was set to 0.05/*M*_eff_, which corresponds to a Bonferroni correction on Meff tests. In other studies of dent and flint maize panels conducted with the same SNP markers, this procedure led to approximately a tenth of the initial number of markers (Rincent et al. 2014). We therefore also considered 0.1*N* as a reference value for comparison. Therefore, three less stringent thresholds, corresponding to 0.1/*M*_eff_, 0.05/0.1 N and 0.10/0.1*N*, were also set (*N* total number of SNPs). All of kinship coefficient (*K*), population structure (*Q*) and principal components analysis (PCA) used in GWAS were estimated in a previous study (Yang et al. 2011) and top 10 axes of variations in PCA were employed in association analysis. The proportion of phenotype variance explained (PVE) by single SNP and all associated SNPs were estimated through a linear regression and corrected for population structure (Q matrix) as follow: *R*^2^ = 1 − RSS1/RSS0 (Xu 2003). RSS0 is the residual sum of squares of a liner regression by population structure to phenotype, and RSS1 is the residual sum of squares of a liner regression by population structure and SNPs (single SNP or all associated SNPs) to phenotype (Xu 2003).

### Linkage mapping

Three F_2:3_ mapping populations were derived from NX531 × SIL8 (NS), TY6 × Mo17 (TM) and TY6 × W138 (TW) with 200, 188 and 284 families, respectively. NX531 and TY6 are two inbred lines with a high KRN, while SIL8, Mo17 and W138 have a low KRN. TY6, Mo17 and W138 were selected from Panel 1 based on the genotype at significant association sites. The number of favorable alleles at the significant association sites in TY6, Mo17 and W138 is listed in Table S3. Genome-wide SSR markers, with 170 for NS, 199 for TM and 184 for TW, were used to genotype the three linkage mapping populations, respectively. The F_2:3_ families of the NS were phenotyped at Xingtai (38°N, 115°E) in 2011; both the TM and TW were phenotyped at Xingtai and Wuhan (30°N, 114°E) in 2012 using a randomized block design with three replicates. The broad-sense heritability was calculated by H_b_^2^ = δ_g_^2^/(δ_g_^2^ + δ_ge_^2^/*n* + δ_e_^2^/*nr*) (δ_g_^2^: genetic variance; δ_ge_^2^: genotype × environment variance; δ_e_^2^: error; *n*: number of environments; *r*: number of replicates). The linkage maps were constructed by MAPMAKER/EXP V3 (Lincoln et al. 1992), and then QTL mapping was conducted under additive and dominant model using the composite interval mapping (CIM) algorithm in the Windows QTL Cartographer 2.5 (Wang et al. 2012) with 5 cM as window size and the threshold LOD = 2.5. A physical region which was repeatedly detected for KRN QTL in different populations was assumed as one common QTL. Previous identified KRN QTLs (Austin and Lee 1996; Veldboom and Lee 1996; Upadyayula et al. 2006; Yan et al. 2006; Liu et al. 2007; Ma et al. 2007; Tang et al. 2010; Tan et al. 2011; Lu et al. 2011a) were collected and projected to the B73 RefGen_v2 genome based on the physical location of flanking makers (Table S6). The frequency of QTL detected repetitively was calculated within a 10 Mb window size sliding every 5 Mb. A genomic region which was detected twice or more was defined as a KRN QTL hotspot.

### Genomic prediction for KRN

**Fig. 1:**
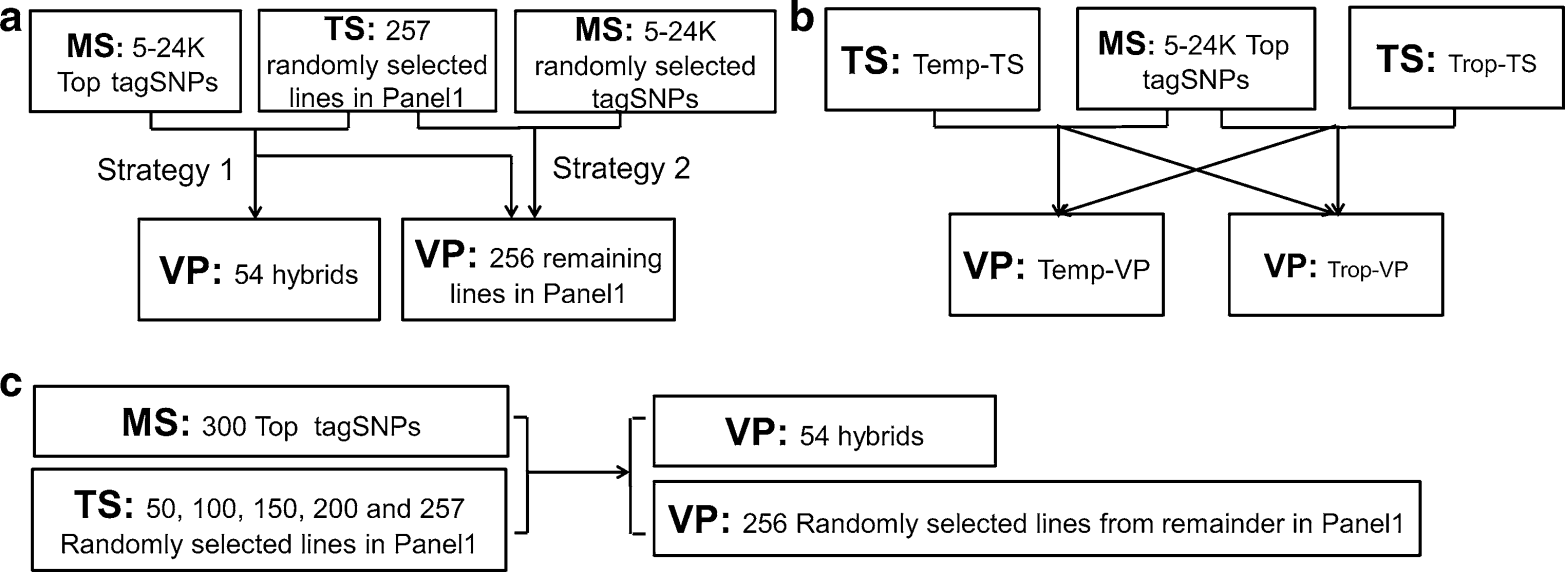
The pipelines of KRN prediction. **a** Prediction of the KRN in Panel 1 and the hybrids using top tagSNPs and randomly selected tagSNPs. **b** The effect of the population structure on genomic prediction. **c** The effect of the TS size on genomic prediction. *MS* marker set, *TS* training set, *VP* validation population, *Temp* temperate lines, *Trop* tropical/subtropical lines

To predict the KRN of the inbred lines and hybrids, we estimated the predictability by whole-genome prediction (WGP). First, the LD among 48,962 SNPs was estimated and was used to classify all of the SNPs to LD blocks based on the threshold *r*^2^ > 0.2. The SNP that was most significantly associated with KRN in a given LD block was labeled as “tagSNP”, and all of the tagSNPs were pooled as a marker bank to sample the marker sets (MSs). Then, we performed a WGP using the ridge-regression best linear unbiased prediction (RR-BLUP) using various MSs, training sets (TSs) and validation populations (VPs) (Piepho 2009; Endelman 2011). To compare effect of the MSs, we adopted two strategies to form the MSs and then to predict the KRN using 257 randomly selected lines (half of Panel 1) as TSs and both the remaining 256 lines in Panel 1 and the 54 single-cross hybrids as VPs (Fig. 1a). These 54 hybrids (Table S2) were produced by biparental crossing among 24 inbred lines, and were phenotyped in 2013 Xingtai (38°N, 115°E). In Strategy 1, five to 24 K tagSNPs (referred to as the Top tagSNPs) were selected according to decreasing by significance [−log(*p* value)]. In Strategy 2, five to 24 K tagSNPs were randomly sampled from the marker bank by automatically incrementing 5 SNPs at the next sampling (Fig. 1a). The predictability of the same size of MS was determined by five repeated samplings. To evaluate the effect of the population structure on the WGP, we classified the lines in Panel 1 into two subpopulations: temperate lines and tropical/subtropical lines. Half of the lines of each subpopulation were randomly selected as TSs (temperate TS: Temp-TS; tropical/subtropical TS: Trop-TS) and the remaining lines as VPs (temperate VP: Temp-VP; tropical/subtropical VP: Trop-VP) (Fig. 1b). Moreover, we selected 300 Top tagSNPs as MS to evaluate the effect of the TS size. The size of the TSs was composed of randomly selected 50, 100, 150, 200, and 257 lines in Panel 1. The 54 hybrids and randomly selected 256 lines from the remainders in Panel 1 were sampled as VPs (Fig. 1c). Each of the above-mentioned prediction procedures was repeated 100 times. The KRN of the inbred lines that was evaluated in each five environments, the BLUP over environments and the KRN of 54 hybrids that were evaluated in 2013 at Xingtai were used as observed values. The correlation (*r*) between the predicted and observed KRN was calculated to evaluate the predictability.

## Results

### Genome-wide association study

The KRN phenotypes of Panel 1 were evaluated under five environments and showed a normal distribution ranging from 9.3 to 19.7 rows per ear, with an average of 13.3 ± 1.6 (Table S3). Importantly, the observed KRN values indicated high repeatability (93.87 %) (Table S3) with significant correlations (*r* = 0.69–0.85) among the five environments (Table S4). As in the study of Yang et al. (2011), Panel 1 can be divided into four subpopulations: Tropical-subtropical (TST), Stiff Stalk (SS), Non-stiff stalk (NSS), and Admix. The KRN of inbred lines in the SS (mid-value 14.0, ranging from 11.1 to 15.7), Admix (mid-value 13.6, ranging from 12.6 to 14.9) and NSS (mid-value 13.3, ranging from 12.3 to 14.2) was higher than that in TST (mid-value 12.8, ranging from 11.8 to 13.6) (Figure S1), indicating the influence of population structure for KRN. About 8.04–26.77 % of KRN variances under different environments could be explained by population structure, indicating that population structure might affect the association analysis of KRN (Table S3).

**Fig. 2:**
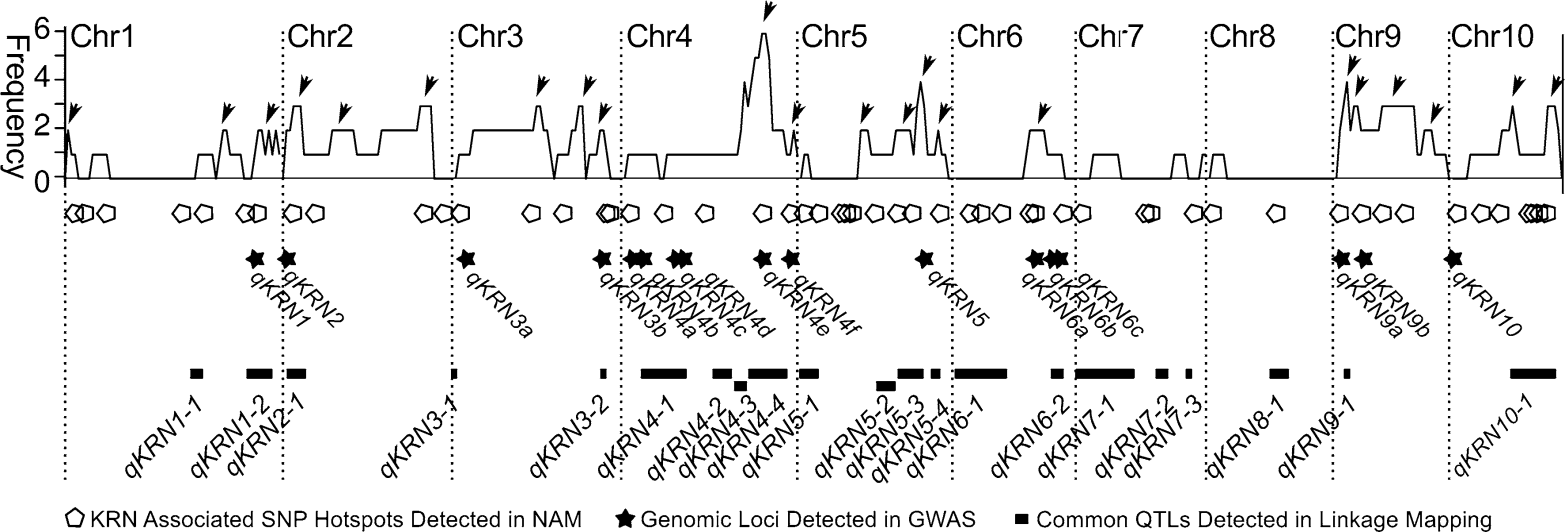
The distribution of genetic loci for KRN detected in this study and previous studies. The *X*-axis represents chromosomes of maize, and the *Y*-axis represents the frequency of KRN QTL repetitively detected on a certain genomic region by previous studies (Table S6). The *black arrowhead* points out the previous identified KRN QTL hotspot. *Pentagons* KRN-associated SNPs hotspots detected in the NAM population (Brown et al. 2011). The *black stars* and *black boxes* represent KRN-associated loci detected in GWAS and common QTLs detected in linkage mapping in this study, respectively

To determine the appropriate model for GWAS, six models (see “Materials and methods”) were evaluated in Panel 1. Compared with GLM model, the three MLM models showed a stronger control for Type I error (Figure S2). In previous studies, the MLM + Q model was successfully applied for GWAS of quantitative traits in Panel 1 (Li et al. 2013; Wen et al. 2014). Therefore, this model was also chosen for GWAS in this study (Figure S2). The number of independent tests (*M*_eff_) for the 48962 SNPs of Panel 1 was 48498.7, similar to total SNPs number. This result was different from the finding of Rincent et al., who found a larger decrease in *M*_eff_ compared to total SNPs number (2014). Genetic diversity in an association population likely influences the number of independent tests. Thus, the estimation of independent tests for various genetic materials deserves further investigation. The 0.05/*M*_eff_ level of Bonferroni corrected −log(*p* value) was 5.99 (Table 1), and the less stringent −log(*p* value) thresholds, corresponding to 0.1/*M*_eff_, 0.05/0.1*N* and 0.10/0.1*N*, were 5.69, 4.99 and 4.69, respectively (Table 1). Under the four thresholds 7, 7, 24 and 31 KRN-associated SNPs were identified, respectively (Table 1). In consideration of possible over correction of the MLM + Q model and narrow KRN variation in Panel 1, the threshold −log(*p* value) 4.69 was used to identify a larger number of KRN-associated SNPs, thereby the 31 KRN-associated SNPs were considered for further investigation. The proportion of phenotype variance explained (PVE) by individual SNP ranged from 2.45 to 10 %, and 24 SNPs had a PVE >5 % (Table S5). In Panel 1, frequency of the allele with positive effect at these 31 SNP sites ranged from 0.09 to 0.79 and was less than 0.3 for 68 % of the sites. Interestingly, frequency of the allele with positive effect at the KRN-associated SNPs was negatively correlated with phenotypic variation explained by these sites (*r* = −0.63, *p* < 0.0001). Considering the criterion of LD with *r*^2^ > 0.2, the 31 SNPs belonged to 17 LD blocks which represented 17 KRN-associated genomic loci (Table 2, Fig. 2) including 4 loci covered by more than one KRN-associated SNP and 13 loci supported by single KRN-associated SNP (Table 2, Table S5). A total 58.8 % (10/17) of KRN-associated genomic loci had a PVE >5 % (Table 2). Furthermore, multiple regressions indicated that 51.10–62.05 % of the phenotypic variation was explained by these 17 genomic loci in the five environments and the BLUP over all environments, respectively (Table S5).

**Table 1:** The number of KRN significant associated SNPs under four thresholds

Threshold^a^	−log(*p* value)	Yaan (2009)	Kunming (2009)	Sanya (2009)	Wuhan (2010)	Kunming (2010)	BLUP	Total
0.1/0.1 N	4.69	5	8	18	3	1	7	31
0.05/0.1 N	4.99	4	7	13	3	0	4	24
0.1/*M*_eff_	5.69	0	2	6	0	0	0	7
0.05/*M*_eff_	5.99	0	2	5	0	0	0	7

*M*_eff_ = 48498.7009 (the number of independent test); *N* = 48962 (the total SNPs number)

^a^The Bonferroni correction thresholds were corrected by the Meff method (Nyholt 2004; Li and Ji 2005)

**Table 2:** Summary of the KRN-associated genomic loci by the GWAS in Panel 1

Genomic loci^a^	SNP Number^b^	Chr^c^	Env^d^	Significant level^e^	PVE (%)^f^	Co-localized QTLs^g^	Co-localization with previous loci^h^
*qKRN1*	1	1	KM2010	4.69	4.75	*qKRN1*-*2*	* #
*qKRN2*	1	2	BLUP	4.69	2.45		#
*qKRN3a*	1	3	YA2009	4.69	4.50		&
*qKRN3b*	2	3	YA2009	4.99	8.16	*qKRN3*-*2*	* #
*qKRN4a*	1	4	WH2010	4.99	2.48		&
*qKRN4b*	4	4	KM&SY2009	4.99	9.30	*qKRN4*-*1*	&
*qKRN4c*	1	4	KM2009	4.99	5.72		&
*qKRN4d*	1	4	WH2010	4.99	3.89		&
*qKRN4e*	10	4	SY2009&BLUP	5.99	9.38	*qKRN4*-*4*	* #
*qKRN4f*	1	4	YA2009	4.99	4.50		* #
*qKRN5*	1	5	SY2009	4.69	9.66		* #
*qKRN6a*	1	6	SY2009	4.69	10.00		#
*qKRN6b*	1	6	SY2009	4.99	6.60	*qKRN6*-*2*	* #
*qKRN6c*	1	6	YA&KM2009	5.99	9.28	*qKRN6*-*2*	* #
*qKRN9a*	1	9	BLUP	4.99	7.00		* #
*qKRN9b*	2	9	KM2009	5.99	9.93		* #
*qKRN10*	1	10	WH2010	4.99	4.79		

^a^Genomic loci were referred by LD SNPs or neighbor SNPs

^b^Associated SNPs number at each genomic locus

^c^Chromosome of the KRN-associated genomic loci

^d^The environments of associated loci detected, *YA* Yaan; *KM* Kunming; *SY* Sanya; *WH* Wuhan

^e^The significant associated thresholds −log(*p* value) of the SNPs in each KRN-associated genomic loci could reach

^f^The maximum phenotype variance explanation (PVE) of SNPs in each KRN-associated loci across five environments and BLUP

^g^Co-localized QTLs detected in NS, TM and TW

^h^Co-localize with previous identified QTLs, “*” represents co-localization with KRN-associated SNPs hotspots in NAM population (Brown et al. 2011); “#” represents co-localization with previous identified KRN QTL hotspots; & represents co-localization with previous identified QTLs (See “Materials and methods”)

### Linkage analysis

Three F_2:3_ populations (NS, TM, and TW) were developed by crossing high KRN lines with low KRN lines to map the KRN QTLs. In each of the F_2:3_ mapping populations, ANOVA analysis also revealed significant genetic variation in the KRN among the F_2:3_ families. The KRN ranged from 10.2 to 17.4 in NS, 10.7 to 18.1 in TM and 10.0 to 19.2 in TW (Table S3, Figure S1). Additionally, the KRN in the TM and TW populations at Wuhan were highly correlated with that at Xingtai (Table S3), and exhibited high broad-sense heritability with 88.39 % in TM and 78.03 % in TW (Table S3). The SSR markers (170 for NS, 199 for TM and 184 for TW) were used for the linkage and QTL mapping. A total of 1836.7 cM-, 2277.7 cM- and 2258.9 cM-length genetic maps were constructed for the NS, TM and TW, with an average distance among the markers of 10.8, 11.4 and 12.3 in NS, TM and TW, respectively. A total of 33 QTLs were detected in the three linkage populations, 7 in the NS, 13 in the TM and 13 in the TW (Table 3). Single QTL explained 2.3–28.4 % of phenotype variance (Table 3), and 94 % (31/33) of QTLs had a PVE >5 % (Table 3). About 63.6 % (21/33) of the QTLs were additive or partially dominant, while 36.4 % (12/33) of the QTLs were completely dominant (Table 3). All dominant loci also displayed largely additive effects. Furthermore, the 33 QTLs were clustered into 21 common QTLs based on the genomic locations of the flanking markers. Of the 21 common QTLs, 12 QTLs had a PVE >5 % and 6 QTLs had a PVE >10 % (Table 3). Five QTLs (*qKRN4*–*3*, *qKRN4*–*4*, *qKRN5*–*1*, *qKRN5*–*4* and *qKRN6*–*2*) were detected across two environments, and four QTLs (*qKRN4*–*4*, *qKRN5*–*1*, *qKRN5*–*4* and *qKRN10*–*1*) were detected in two or more populations (Table 3).

**Table 3:** KRN QTLs those were detected in three F_2:3_ families

QTL	Env	Pop	Chr	Flanking markers	Genetic interval cM	Physical interval Mb	LOD	A	D	PVE (%)	d/a^a^	Gene action
*qKRN1*–*1*	2012 XT	TM	1	umc2396-bnlg1025	36	24.4	7.83	0.73	−0.29	9.67	0.39	pd
*qKRN1*–*2*	2012 XT	TW	1	umc1184-M1.108	12.7	37.6	2.77	−0.4	0.39	2.49	0.97	d
*qKRN2*–*1*	2010 XT	NS	2	bnlg1537-prp2	27.9	24.3	6.07	0.56	0.06	8.82	0.11	a
*qKRN3*–*1*	2010 XT	NS	3	phi37411-bnlg1325	30.7	4	6.68	0.55	0.04	8.44	0.07	a
*qKRN3*–*2*	2012 XT	TW	3	umc2152-umc2048	22.4	9.9	4.87	0.41	−0.03	4.21	0.07	a
*qKRN4*–*1*	2012 XT	TW	4	umc1117-umc1791	12.7	71.1	2.77	0.2	0.24	2.33	1.21	d
*qKRN4*–*2*	2012 XT	TW	4	umc1346-umc1137	13.6	26.8	2.95	0.31	0.26	3.04	0.84	d
*qKRN4*–*3*	2012 WH	TM	4	umc1329-umc1194	27.5	14.4	5.98	0.49	0.1	12.67	0.21	a
*qKRN4*–*3*	2012 XT	TM	4	bnlg1137-umc1086	58.1	22.8	12.63	0.71	0.11	18.54	0.16	a
*qKRN4*–*4*	2012 XT	TW	4	umc1194-umc2188	40.4	30.6	26.65	1.24	−0.09	26.05	0.08	a
*qKRN4*–*4*	2012 WH	TW	4	umc1194-umc2188	54.3	30.6	11.8	0.91	−0.01	18.34	0.02	a
*qKRN4*–*4*	2010 XT	NS	4	bnlg2162-umc1284	63.6	49.7	13.82	0.78	0.07	20.71	0.09	a
*qKRN5*–*1*	2012 WH	TM	5	umc1097-umc2578	18	17.9	3.92	0.17	0.27	7.31	1.55	d
*qKRN5*–*1*	2010 XT	NS	5	umc1365-umc1464	66.7	8.9	14.49	1.2	−0.17	28.43	0.14	a
*qKRN5*–*1*	2012 XT	TW	5	umc2036-umc2578	39.1	13.2	8.51	0.36	0.34	7.71	0.93	d
*qKRN5*–*1*	2012 XT	TM	5	umc1587-umc1056	19.5	21.5	4.25	0.41	−0.04	5.51	0.11	a
*qKRN5*–*1*	2012 WH	TW	5	umc1894-umc1056	29.2	19.8	6.35	0.45	0.05	7.4	0.1	a
*qKRN5*–*2*	2012 WH	TW	5	umc1056-umc1389	26.1	36.7	5.68	0.38	0.2	7.33	0.54	pd
*qKRN5*–*3*	2012 XT	TM	5	umc1171-bnlg1306	38.3	38.7	8.32	0.78	−0.26	11.78	0.34	pd
*qKRN5*–*4*	2012 WH	TW	5	umc1941-umc1072	14.1	19.2	3.06	0.46	−0.05	5.48	0.11	a
*qKRN5*–*4*	2012 XT	TW	5	umc1941-umc1072	25.2	19.2	5.49	0.57	−0.14	6.71	0.25	a
*qKRN5*–*4*	2012 WH	TM	5	bnlg1306-umc1072	17.2	2.6	3.74	0.6	−0.33	7.79	0.55	pd
*qKRN5*–*4*	2012 XT	TM	5	bnlg1306-umc1072	11.9	2.6	2.58	0.66	−0.23	4.11	0.34	pd
*qKRN6*–*1*	2012 XT	TW	6	umc1143-umc1595	45.6	91.9	9.91	0.53	0.18	8.44	0.34	pd
*qKRN6*–*2*	2012 XT	TM	6	umc1020-umc1296	34	15.4	7.4	0.45	0.26	9.74	0.58	pd
*qKRN6*–*2*	2012 WH	TM	6	umc1859-umc1248	20.8	14.8	4.53	0.24	0.4	9.96	1.66	d
*qKRN7*–*1*	2012 XT	TM	7	mmc0171-umc1929	13.9	103.9	3.01	−0.29	0.5	3.34	1.71	d
*qKRN7*–*2*	2010 XT	NS	7	umc1125-umc1407	12.7	3.4	2.76	0.26	0.27	4.46	1.03	d
*qKRN7*–*3*	2012 XT	TM	7	umc1983-bnlg1022	12.5	23.5	2.71	−0.27	0.54	3.66	1.99	d
*qKRN8*–*1*	2010 XT	NS	8	bnlg2181-umc2199	15.1	26.7	3.29	0.39	−0.57	5.39	1.47	d
*qKRN9*–*1*	2012 XT	TM	9	umc1170-umc1037	12.6	6.1	2.74	−0.01	0.48	4.38	48	d
*qKRN10*–*1*	2010 XT	NS	10	bnlg1526-umc1506	20.3	15.4	4.4	0.05	0.42	4.96	7.91	d
*qKRN10*–*1*	2012 WH	TW	10	umc1995-umc1640	19.5	56	4.24	0.65	0.33	15.51	0.51	pd

*ENV* Environment, *XT* Xingtai, *WH* Wuhan, *Pop* population, *Chr* chromosome, *A* Additive effect, *D* Dominant effect, *PVE* phenotype variance that was explained, *pd* partial dominant effect, *d* dominant effect

^a^The degree of dominance, d/a (dominant effect/additive effect) (Stuber et al. 1987)

### Co-localization between QTL and associated genomic loci for KRN

The flanking markers of common QTLs detected in this study were projected to the B73 RefGen_v2 to anchor the QTL physical location. We found that 61.3 % (19/31) of the KRN-associated SNPs or 35 % (6/17) of the KRN-associated genomic loci (*qKRN1*, *qKRN3b*, *qKRN4b*, *qKRN4e*, *qKRN6b* and *qKRN6c*) co-localized with 5 common QTLs (Table 2; Fig. 2). Furthermore, 75 KRN QTLs and 261 KRN-associated SNPs that were identified in previous studies were collected and were then projected to the B73 RefGen_v2. This projection resulted in 22 KRN QTL hotspots and 50 KRN-associated SNPs hotspots (Fig. 2). A total of 65 % (11/17) KRN-associated genome loci fell into the KRN QTL hotspots, among these, five co-localized with QTL regions identified in previous studies (Fig. 2; Table 2). Nine KRN-associated genomic loci co-localized with KRN-associated SNP hotspots which were detected in NAM population (Fig. 2; Table 2). Resultantly, 70 % (12/17) KRN-associated genomic loci were cross-validated by linkage mapping QTLs in this study, as well as by KRN QTL hotspots and KRN-associated SNPs in earlier investigations (Fig. 2; Table 2).

### Genomic prediction for KRN

**Fig. 3:**
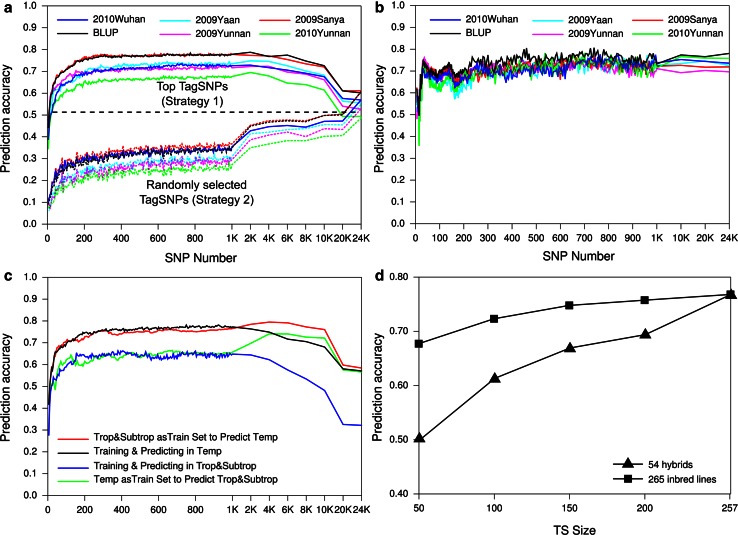
Predictability using tagSNPs for the kernel row number. **a** Predictability of the top tagSNPs and randomly selected tagSNPs in the *inbred lines*. *Continuous lines* the prediction accuracies using 5–24 K top tagSNPs (strategy 1); *Dotted lines* the prediction accuracies using 5–24 K randomly selected tagSNPs (strategy 2). **b** Prediction accuracies using 5–24 K top tagSNPs for 54 hybrids. **c** Prediction accuracies of 5–24 K top tagSNPs in different subpopulations using different training sets and validation populations. **d** Predictability of different sizes of training sets using 300 top tagSNPs in inbred *lines* and *hybrids*

According to the LD value, 48,962 SNPs with MAF >5 % were grouped into 24,521 LD blocks which were represented by 24,521 tagSNPs to generate MSs (marker sets). Using the MS comprising of 17 SNPs, which represented 31 KRN-associated SNPs in the GWAS (Table 4), the prediction accuracies of the KRN were 0.48–0.60 for the inbred lines and 0.64–0.69 for the hybrids (Fig. 3a, b). When the MSs increased from 5 to 300 top tagSNPs, the prediction accuracies of the inbred lines and hybrids increased sharply and reached to the highest level (*r* = 0.78) (Fig. 3a, b; Table 4), while the prediction accuracies of the inbred lines decreased when more than 2 K top tagSNPs were used. In addition, the prediction accuracies also increased quickly when 5–200 randomly selected tagSNPs were used, increased slowly when the tagSNPs increased from 200 to 1 K (*r* < 0.35), and rapidly increased again when the randomly selected tagSNPs increased from 1 K to 24 K (Fig. 3a; Table 4). These results suggest that the prediction accuracy of the randomly selected tagSNPs was lower than that of the top tagSNPs, and that a small subset of markers (approximately 300 top tagSNPs) might be required for the KRN prediction of the inbred lines; additional markers could not improve the predictability. For the KRN prediction of the hybrids, a higher prediction accuracy (*r* = 0.76) was observed when ~40 top tagSNPs were used and was maintained at a high level along with increasing the top tagSNPs (Fig. 3c; Table 4), indicating that the KRN of the hybrids can be predicted by the additive effect of the SNPs which were estimated using inbred lines as a training set.

**Table 4:** The prediction accuracies (%) using the top tagSNPs and randomly selected tagSNPs for the inbred lines and hybrids

SNP number	Prediction	2009 (Yaan)	2009 (Kunming)	2009 (Sanya)	2010 (Kunming)	2010 (Wuhan)	BLUP
17	Strategy 1 for Panel 1	53	60	59	48	49	57
	Strategy 1 for Hybrids	64	65	69	59	64	68
	Strategy 2 for Panel 1	9	10	15	9	14	13
300	Strategy 1 for Panel 1	73	71	78	66	70	77
	Strategy 1 for Hybrids	71	69	67	75	69	71
	Strategy 2 for Panel 1	27	26	33	22	31	31
1 K	Strategy 1 for Panel 1	73	72	78	68	73	78
	Strategy 1 for Hybrids	73	71	72	71	74	73
	Strategy 2 for Panel 1	31	29	37	27	35	35
10 K	Strategy 1 for Panel 1	68	66	72	61	68	73
	Strategy 1 for Hybrids	73	69	73	77	75	77
	Strategy 2 for Panel 1	46	44	50	40	47	50
20 K	Strategy 1 for Panel 1	56	54	61	49	58	61
	Strategy 1 for Hybrids	75	70	72	76	74	77
	Strategy 2 for Panel 1	45	43	50	41	47	50
24 K	Strategy 1 for Panel 1	56	53	61	49	57	60
	Strategy 1 for Hybrids	73	70	72	76	74	78
	Strategy 2 for Panel 1	55	53	61	49	57	61

Strategy 1 for Panel 1: using the top tagSNPs to predict the inbred lines in Panel 1; Strategy 1 for Hybrids: using the top tagSNPs to predict the hybrids; Strategy 2 for Panel 1: using the randomly selected tagSNPs to predict the inbred lines in Panel 1

To evaluate the effect of both the TS and VP in the WGP, we compared the predictability of various combinations between the TS and VP. First, when the TSs and VPs were divided by population structure (Temp-TS, Temp-VP, Trop-TS and Trop-VP), the prediction accuracies increased sharply with the increasing number of the top tagSNPs from 5 to 300 and decreased with the increasing number of the top tagSNPs from 1 K to 24 K. For the temp-VP or trop-VP, the prediction accuracies were almost the same when the temp-TS and trop-TS were used, respectively. Moreover, no matter whether the temp-TS or trop-TS was used, the prediction accuracies were always higher for the temp-VP than for the trop-VP. Second, with the increasing TS size, the accuracies increased slowly in the prediction of the inbred lines but increased quickly in the prediction of the hybrids (Figs. 1c, 3d), demonstrating that the TS size affects the predictability of whole-genome prediction, consistent with the report of Riedelsheimer et al. (2013).

## Discussion

### Genetic architecture of the maize kernel row number

Since the first application of molecular marker technology in QTL mapping in the 1980s, numerous QTLs for agriculturally important traits have been identified in diverse populations. The QTLs for KRN have been widely assayed, and hundreds of QTLs have been identified (Veldboom and Lee 1994; Austin and Lee 1996; Ribaut et al. 1997; Yan et al. 2006; Lu et al. 2011a; Li et al. 2014). In general, investigations using segregation populations that were derived from biparental cross, such as RILs, BCn, and DH, frequently identified only few major QTLs (~10 % PVE) and several minor QTLs due to limited allelic effect differences between the two parents. Evidence from the GWAS in the maize NAM population indicates that the flowering time (Buckler et al. 2009), resistance to southern leaf blight (Poland et al. 2011) and leaf architecture (Tian et al. 2011) are dominated by small additive QTLs with few genetic or environmental interactions. In this study, KRN of maize exhibited a high repeatability and a relatively narrow variation range, and was influenced by population structure in Panel 1. By GWAS, we found about 51.10–62.05 % KRN variance in Panel 1 was dominated by 17 genomic loci, of which 10 loci had a PVE >5 % (Table 2, Table S5). In Panel 1, frequency of positive allele of the KRN-associated SNPs ranged from 0.09 to 0.79, was negatively correlated with its PVE, and most of SNP sites (68 %) had a low frequency of positive allele (<0.3) (Table S5), suggesting that those SNPs with low frequency seem to have a large genetic effect for KRN. A similar result was also observed by Brown et al. (2011), who determined that those loci for inflorescence traits have larger effects than do the flowering- and leaf trait-associated loci, and these large effect alleles had low frequency. This result indicated that favorable alleles of the KRN-associated loci were held by a few inbred lines in Panel 1. As described by Yang et al. (2011), Panel 1 is composed of a set of elite inbred lines, including parental lines of high vigor hybrids, inbred lines utilizing in maize breeding program, and improving lines from the germplasm enhancement of maize project (GEM). A low frequency of positive allele at the KRN-associated SNPs in Panel 1 implicated that favorable KRN alleles have not yet been fully integrated into elite inbred lines. Therefore, those KRN-associated SNPs and QTLs detected in this study give us target loci for improving KRN of elite inbred lines of maize.

The consistency between the association loci from the GWAS and QTLs from linkage mapping provides a cross-validation of the mapping results from the two approaches and also indicates the important loci for the KRN. In this study, three linkage mapping populations were used to validate the GWAS results. We found that ~70 % of the KRN-associated genomic loci detected in this study were cross-validated by KRN QTLs identified in the three F_2:3_ families, KRN QTLs hotspots observed in previous studies and KRN-associated SNP hotspots detected in NAM population (Fig. 2; Table 2). A total of 48 % of common QTLs were proved to be consistent with previously identified QTLs hotspots (Fig. 2). Integrating the KRN QTLs that were detected in the previous and present studies by linkage and GWAS, we found more than 40 loci, comprising some of the large additive loci (>3 %) and many small additive loci (<3 %), which dominate the natural variation in the KRN in maize.

On the basis of the degree of dominance (d/a), QTLs that were detected in the linkage populations were divided into three types: additive, partial dominant, and dominant (Stuber et al. 1987). Of the 33 QTLs that were detected in this study, 13 QTLs (39.4 %) were additive, 8 QTLs (24.2 %) were partially dominant and 12 QTLs (36.4 %) were dominant or overdominant. A total of 6 of the 13 additive QTLs (46.2 %) had a PVE >10 %, ranging from 12.67 to 26.05 %, while 4 of the 12 partial dominant QTLs (33.3 %) had a PVE close to or greater than 10 %, ranging from 9.67 to 15.51 %. Conversely, only one dominant QTL showed a PVE close to 10 % (Table 3), clearly indicating that QTL with additive and partial dominant effect play a major role in the genetic architecture of the KRN.

### Whole-genome prediction of the kernel row number

One of the objectives of the identification of genetic loci is to use those loci to guide the improvement of important traits in maize hybrid breeding. Brown et al. (2011) suggested that an additive model showed a low predictive ability for KRN in NAM. However, in this study, we found that the prediction accuracies of the KRN in the maize inbred lines and hybrids were higher (*r* = 0.66–0.78) than in the NAM population (Brown et al. 2011) and were similar to the predictability in the DH and RIL lines (Riedelsheimer et al. 2013; Guo et al. 2013). Particularly, a high predictability could be achieved by selecting a small subset of top tagSNPs (100–300 top tagSNPs). A similar conclusion was also observed in the NAM population for ear trait prediction (Brown et al. 2011), demonstrating the importance of trait-associated loci (SNPs) for WGP. Earlier studies have suggested that the proportion of genetic variance that is explained by the SNPs is important for the accuracy of the WGP (Campos et al. 2010; Riedelsheimer et al. 2013). For 17 of the 300 top tagSNPs that were detected in the GWAS, the prediction accuracies were 0.48–0.60 for the inbred lines and 0.64–0.69 for the hybrids. The predictability could be improved by 18 % by the other 283 top tagSNPs that failed to be detected in the GWAS. These results also support the hypothesis that the maize KRN in natural population is controlled by some of large additive loci and many of minor additive loci. In addition, the additive model that was used in this study showed a high predictability for the KRN of inbred lines and hybrids, indicating that the KRN might be mainly controlled by additive loci.

Compared with the randomly selected tagSNPs, the top tagSNPs could effectively improve the prediction accuracy (Fig. 2a). Interestingly, when the number of top tagSNPs was increased to more than 1 K, the predictability for the KRN of inbred lines in Panel 1 decreased to *R*^2^ < 0.65, which could be obtained by selecting just <50 top tagSNPs (Fig. 3a). This result demonstrates that a small subset of the trait-associated markers is required for the KRN prediction of inbred lines and randomly high-density SNPs as a marker set cannot improve the predictability in the WGP. A possible interpretation is that numerous neutral SNPs, which are not associated with a trait, cannot genetically improve the proportion of the variance that is explained by SNPs. Therefore, a small subset of top tagSNPs, which can effectively save cost and time, should be recommended for the WGP of the KRN in maize molecular breeding.

Both size of TS and relatedness between TS and VP, which strongly influence the accuracy of SNP effects, are important for predictability in genomic selection (Clark et al. 2012; Riedelsheimer et al. 2013). For natural population, population structure is also a key factor for dissecting genetic architecture (Rafalski 2010). Inbred lines in Panel 1 could be divided by the population structure and adaptiveness into temperate and tropical and subtropical lines. Although temperate lines and tropical and subtropical lines show high genetic divergence (Lanza et al. 1997; Lu et al. 2011b), the prediction accuracies for the temp-VP or trop-VP were almost the same no matter whether temp-TS or trop-TS was used (Fig. 3c). However, the prediction accuracies for trop-VP were lower than that for temp-VP (Fig. 3d). Two main factors might result in the decline of predictive ability for trop-VP: (1) the rapid LD decay in tropical and subtropical lines attenuated the relationship between LD tagSNPs and causal variants, led to more false positive SNPs detected which, in turn, strongly reduced the predictive ability (Lu et al. 2011a; Campos et al. 2010). (2) The greater variability among tropical and subtropical lines relative to temperate lines (Lanza et al. 1997) might affect the accuracy of effects of causal variants. In addition, we found that the prediction accuracies were highly positively correlated with the TS size in this study (Fig. 3d), indicating a large sample of the TS is required for the WGP.

In conclusion, by GWAS in Panel 1, we identified 31 KRN-associated SNPs which represented 17 KRN-associated genomic loci. In the 17 KRN-associated loci, 16 loci were validated by 21 common QTLs which were identified from three linkage populations (TM, TW and NS) and 22 KRN QTL hotspots identified in previous studies. Although none of variations in these loci were confirmed to be the causal variations for KRN, those Top tagSNPs from GWAS were successfully employed to predict KRN of inbred lines and hybrids using RR-BLUP. Moreover, we found a high predictability can be achieved through selecting hundreds of the Top tagSNPs in inbred lines and hybrids, suggesting the Top tagSNPs should be potential target loci for KRN improvement with genomic selection in maize breeding.

#### Author contribution statement

LL, JY, and ZZ carried out the GWAS of the KRN in Panel 1 and Panel 2 and drafted the manuscript. LL, YD, DH, MW, XC and XS participated in the linkage mapping. LL, ZZ and YZ participated in the design of the study and the interpretation of the results and wrote and edited the manuscript.

## Electronic supplementary material

Supplementary material 1 (TIFF 456 kb)

Supplementary material 2 (TIFF 173 kb)

Supplementary material 3 (TIFF 692 kb)

Supplementary material 4 (XLSX 23 kb)

Supplementary material 5 (XLSX 10 kb)

Supplementary material 6 (XLS 27 kb)

Supplementary material 7 (XLS 23 kb)

Supplementary material 8 (XLSX 16 kb)

Supplementary material 9 (XLSX 13 kb)
