# Supplementary figures and images for: Genetic architecture of maize kernel row number and whole genome prediction
Source: Theor Appl Genet. 2015 Jul 19;128(11):2243–54. doi: 10.1007/s00122-015-2581-2 (PMC4624828; doi:10.1007/s00122-015-2581-2)

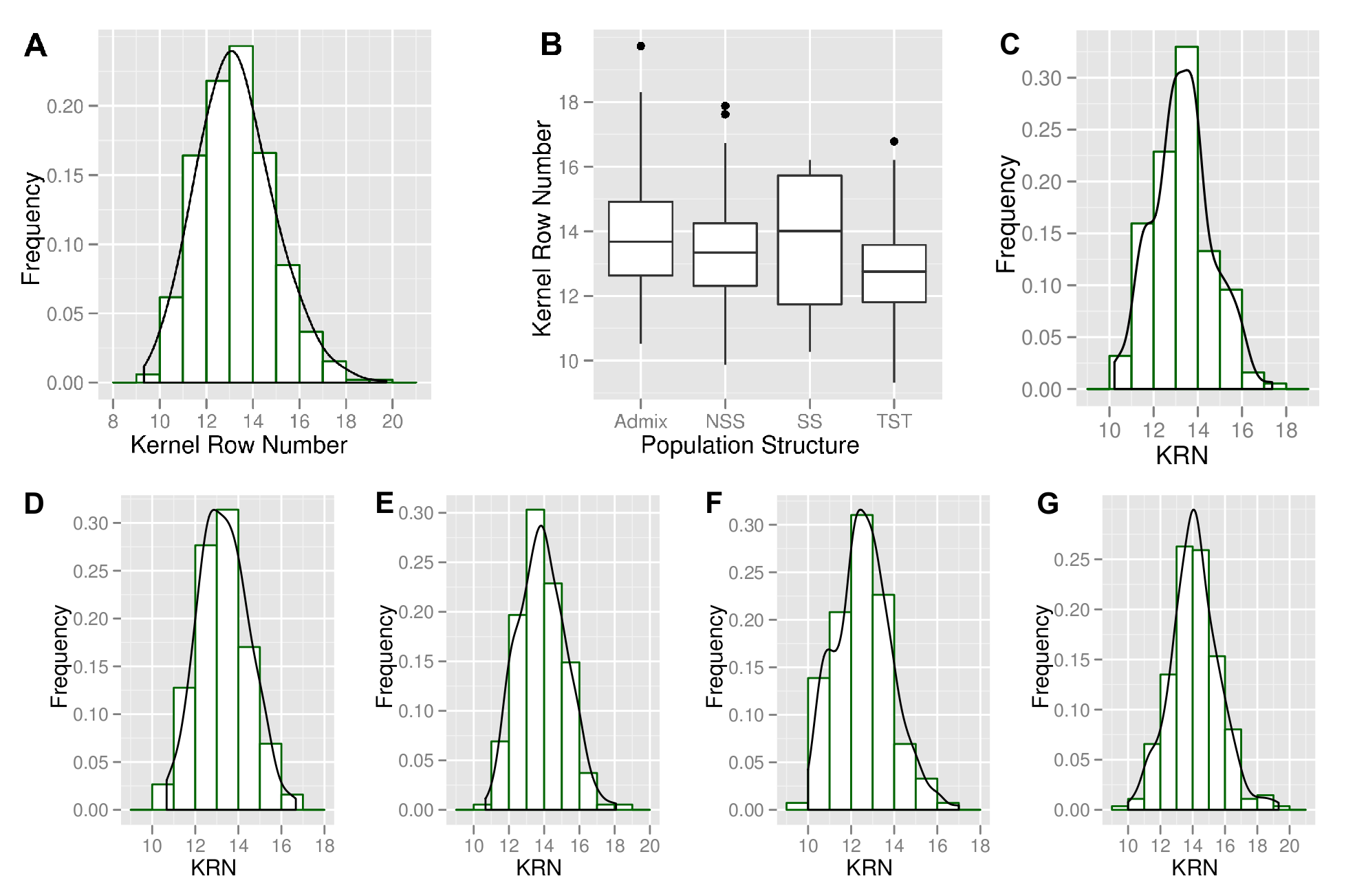

Supplement: Supplementary file 1 — Supplementary material 1 (TIFF 456 kb) [file 122_2015_2581_MOESM1_ESM.tiff]

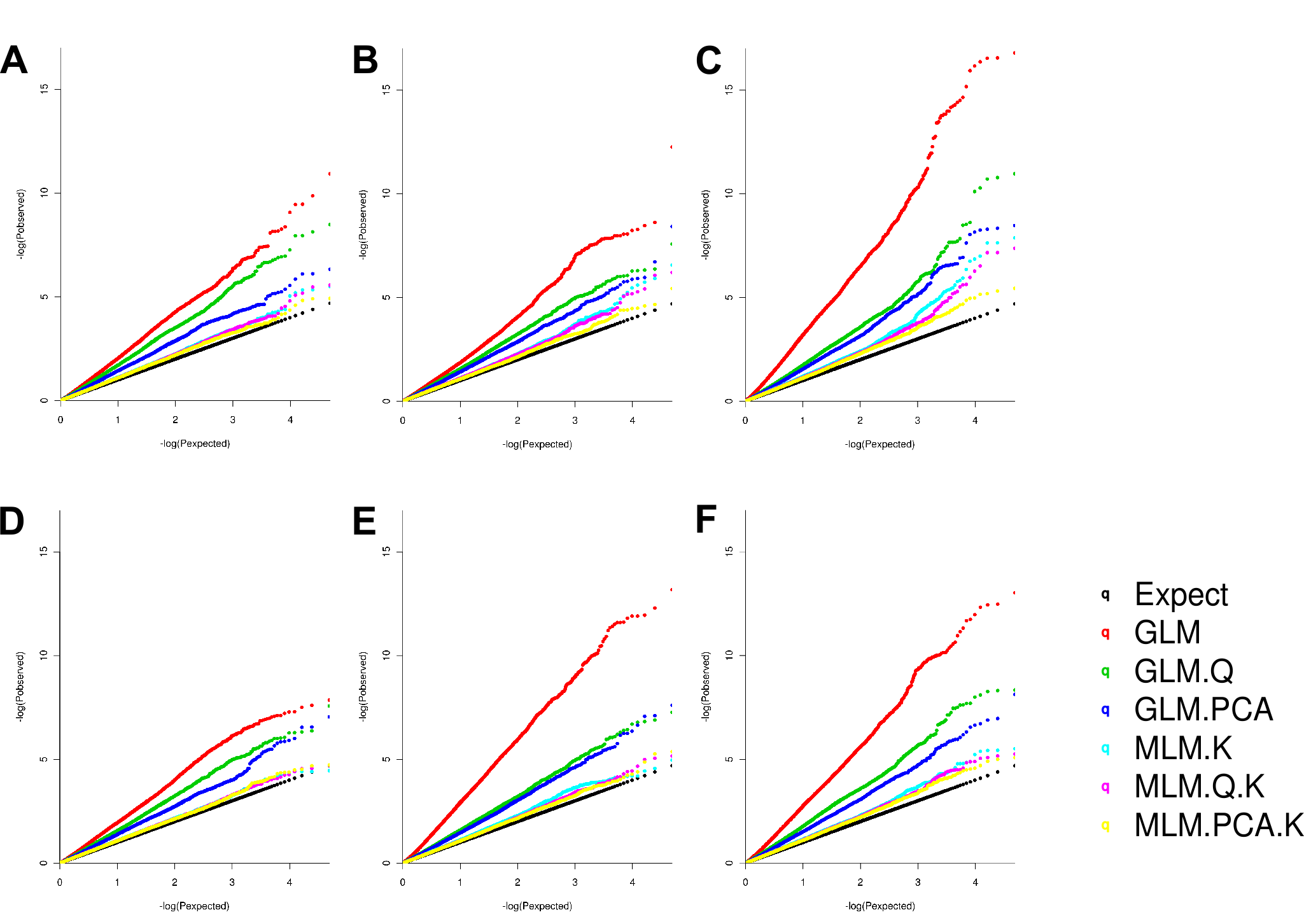

Supplement: Supplementary file 2 — Supplementary material 2 (TIFF 173 kb) [file 122_2015_2581_MOESM2_ESM.tiff]

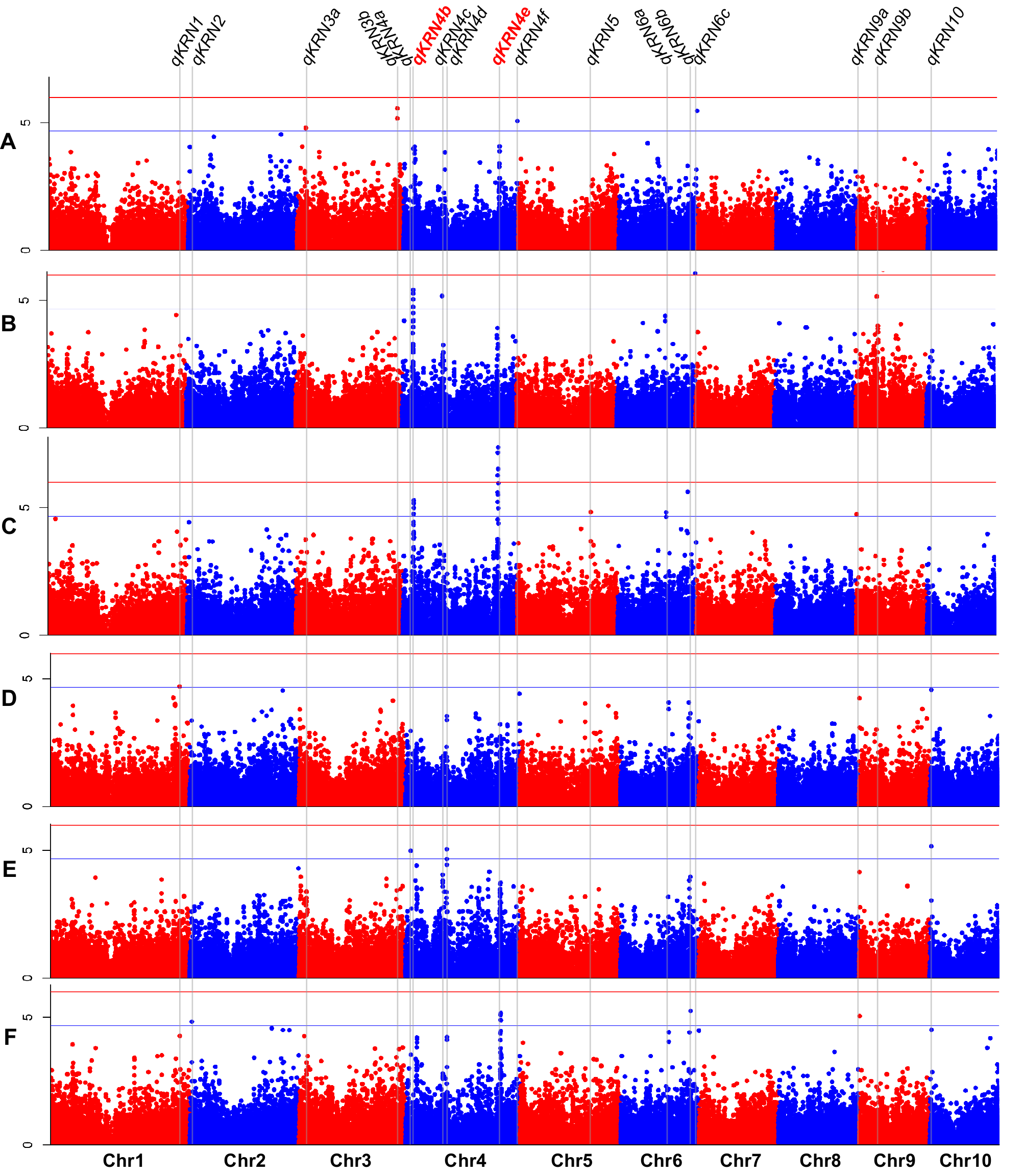

Supplement: Supplementary file 3 — Supplementary material 3 (TIFF 692 kb) [file 122_2015_2581_MOESM3_ESM.tiff]
